# Pilot genome-wide association study of antibody response to inactivated SARS-CoV-2 vaccines

**DOI:** 10.3389/fimmu.2022.1054147

**Published:** 2022-11-14

**Authors:** Ping Li, Dawei Shi, Wenlong Shen, Shu Shi, Xinjie Guo, Jia Li, Sihong Xu, Yan Zhang, Zhihu Zhao

**Affiliations:** ^1^ Department of Protein Engineering, Beijing Institute of Biotechnology, Beijing, China; ^2^ Division II of In Vitro Diagnostics for Infectious Diseases, Institute for In Vitro Diagnostics Control, National Institutes for Food and Drug Control, Beijing, China; ^3^ Division of Arboviral Vaccine, National Institutes for Food and Drug Control, Beijing, China

**Keywords:** COVID-19, SARS-CoV-2, vaccine, antibody, SNP, GWAS

## Abstract

Vaccines are a key weapon against the COVID-19 pandemic caused by SARS-CoV-2. However, there are inter-individual differences in immune response to SARS-CoV-2 vaccines and genetic contributions to these differences have barely been investigated. Here, we performed genome-wide association study (GWAS) of antibody levels in 168 inactivated SARS-CoV-2 vaccine recipients. A total of 177 SNPs, corresponding to 41 independent loci, were identified to be associated with IgG, total antibodies or neutral antibodies. Specifically, the rs4543780, the intronic variant of FAM89A gene, was associated with total antibodies level and was annotated as a potential regulatory variant affecting gene expression of FAM89A, a biomarker differentiating bacterial from viral infections in febrile children. These findings might advance our knowledge of the molecular mechanisms driving immunity to SARS-CoV-2 vaccine.

## Introduction

Coronavirus disease 2019 (COVID-19) is an infectious disease caused by Severe Acute Respiratory Syndrome-Coronavirus 2 (SARS-CoV-2). It was first discovered in 2019 (1–3) and has spread worldwide thereafter, resulting more than 610 million infections and six million deaths up to September 21, 2022 (https://covid19.who.int/). Therefore, the development of safe and effective vaccines against SARS-CoV-2 has been urgently needed.

An inactivated vaccine is one that uses a killed pathogen to stimulate the immune system to protect the body against infection, which has been successfully applied in preventing diseases such as polio, hepatitis A, influenza, Japanese encephalitis and rabies (4). Two kinds of inactivated SARS-CoV-2 vaccines, BBIBP-CorV and CoronaVac (5, 6), developed by Sinopharm-Bejing Institute of Biological Products Co. and Sinovac Life Sciences respectively, have shown safety and efficiency in clinical trials (7–10) and were granted for emergency use by the World Health Organization. Antibodies induced by vaccines play a key role in preventing disease, and researches indicate that neutralizing antibody levels are highly predictive of immune protection from symptomatic SARS-CoV-2 infection (11). However, differential antibody response to SARS-CoV-2 vaccines in healthy subjects is observed (12) while the related factors remain to be defined.

Human genetic background has been suggested to contribute to inter-individual difference in antibody response to many vaccines (13, 14). For example, multiple studies revealed HLA variants were associated with antibody response to hepatitis B vaccine (15–18). CD46 and IFI44L genetic variants were revealed to be associated with neutralizing antibody response to measles vaccine (19). Common SNPs in IL18R1 and IL18 genes were associated with variations in humoral immunity to smallpox vaccination in both Caucasians and African Americans (20). In addition, recent studies suggested that SNPs in the regulatory region of IGH gene were associated with antibody levels in response to SARS-CoV-2 vaccine (21). However, a genome-wide profiling of genetic variants associated with the antibody levels induced by the SARS-CoV-2 vaccine is still lacking.

Here, we performed genome-wide association study (GWAS) of genetic variants associated with antibody levels induced by inactivated SARS-CoV-2 vaccines. A total of 117 SNPs, corresponding to 41 independent loci, were identified to be associated with IgG, total antibodies (Ab) or neutral antibodies (NAbs) (P < 5e-7). Specifically, the rs4543780, residing in the intron of FAM89A (Family With Sequence Similarity 89 Member A) gene, which was associated with total antibodies level (P = 2.86e-7), was annotated as a potential regulatory variant affecting FAM89A gene expression. These findings might advance our knowledge of the precise mechanisms driving immunity to SARS-CoV-2 vaccine.

## Materials and methods

### Study participants

A total of 176 individuals who received two doses of SARS-CoV-2 inactivated vaccine was recruited at Beijing, China between February 24th and June 25th, 2021. The inactivated vaccine was either BBIBP-CorV (Sinopharm and Bejing Institute of Biological Products Co., Beijing, China) or CoronaVac (Sinovac Life Sciences, Beijing, China). After removing close related individuals and poor genotyped individuals based on genome-wide genotyping data, 168 individuals were kept for further analysis. Informed consent was obtained from all vaccinated volunteers enrolled in studies at the Beijing BGI Clinical Laboratories. The Institutional Review Board (IRB) of BGI-Shenzhen approved the serological and genomic polymorphism analyses of samples collected by the aforementioned institution under ethical clearance No. BGI-IRB 20158.

### Antibody level assay

Serum samples were collected between day 12 and day 141 after the second dose of vaccine to measure antibody levels. IgG, IgA, IgM, total antibodies and neutralizing antibodies against SARS-CoV-2 were detected using magnetic chemiluminescence enzyme immunoassay kits (Bioscience), according to the manufacturer’s instructions. Antibody levels are presented as the measured chemiluminescence values divided by the cutoff (absorbance/cutoff, S/CO). The cutoff value of this test was defined by receiver operating characteristic curves. An S/CO value higher than 1 was regarded as positive.

### Genotyping, imputation and quality control

Genomic DNAs were extracted from 200uL of peripheral whole blood, according to the manufacturer’s instructions (CWBIO, Magbead Blood DNA Kit). The DNA concentration was measured using Nanodrop, and the DNA degradation and contamination were monitored in 1% agarose gels.

Genotyping was performed using the CBT_PMRA Array, consisted of about 0.8 million SNPs, at CapitalBio Corporation (Beijing, China). Two of 176 samples had low dish quality control (DQC < 0.82) and were removed from further genotype calling. Genotype callings were performed using Axiom Analysis Suite 3.1.51 based on the default workflow. All the 174 individuals had genotype call rates > 90%. Seven individuals showed sex discrepancies and were changed of sex assignments to those imputed from X chromosome inbreeding coefficients. Six individuals were excluded as they were related with the other individuals based on pairwise identity-by-state by “PI_HAT” values in PLINK 1.9 (PI_HAT > 0.5 and between 0.25 and 0.5 indicates the first- and second-degree relatives, respectively), leaving 168 individuals for further analysis.

Imputation on the genotyping data of chromosomes 1-22 was performed using the ChinaMAP Imputation Server (http://www.mbiobank.com/imputation/) (22), a genotype imputation server utilizing the ChinaMAP reference panel constructed from the China Metabolic Analytics Project (ChinaMAP) WGS dataset. After imputation, the SNPs with R-squares (R^2^) below 0.6 or minor allele frequencies (MAF) < 0.01 were excluded. Further SNP quality controls filtered out SNPs that had call rates < 90%, deviated significantly from Hardy–Weinberg equilibrium (HWE) (P < 1e-6) or MAF < 0.05. Finally, a total of 5,089,908 SNPs passed quality controls.

### Genome-wide association tests

Genetic association analysis was conducted using PLINK 1.9 software (23). As for IgG, Ab or NAbs levels, we carried out genome-wide association tests in linear regression models. As for IgA or IgM level, we categorized the antibody level as two group: S/CO ≥ 1 as positive group and S/CO < 1 as negative group, and carried out genome-wide association tests in logistic regression models. Either linear regression or logistic regression analysis adjusted for covariates including: age, gender, vaccine type, time from the 2nd dose of immunization to blood draw, and the top six principal components from PCA to correct for population stratification. To display the association results, Manhattan plots were constructed using the R-package qqman (24). Quantile–quantile (Q-Q) plots of the observed -log10 (P) against values predicted from the reference distribution under the null hypothesis were constructed and values of lambda (λ) inflation factor were calculated to assess any inflation in the levels of significance (25).

### Annotation of significant SNPs

The position of significant SNPs relative to genes was annotated by Ensembl Variant Effect Predictor (VEP) (26). Regulatory potential of significant SNPs was prioritized using RegulomeDB (27), a database that annotates SNPs based on chromatin immunoprecipitation (ChIP)-seq, formaldehyde-assisted isolation of regulatory elements (FAIRE), and DNase I hypersensitive site data sets from the Encyclopedia of DNA Elements (ENCODE) project (28). Regional plot of significant SNPs were performed by LocusZoom (29).

### Statistical analysis

The antibody levels in different vaccine type groups were compared using Kruskal-Wallis rank sum test. Comparing antibody levels between males and females was conducted using Wilcoxon rank sum test with continuity correction. Pearson correlation test was used to examine correlation of levels of different antibodies, as well as correlation of antibody levels with age and the interval from the 2nd dose of vaccine to blood draw. P < 0.05 was considered as statistically significant.

The currently accepted P-value threshold to declare a SNP to be genome-wide significant is < 5e-8. Because our current study is comprised of limited number of subjects, and because we plan to identify more candidate SNPs to be further validated and studied, we have chosen the less stringent, but still conservative significance threshold of 5e-7 and considered variants with P > 5e-8 but < 5e-7 as suggestive evidence of association to be highlighted in this report as well. Power analysis by Genetic Power Calculator (30) indicated that for a variant with a MAF of 0.2 and a heritability of 2% and given type I error rate of 5e-7, the sample size to achieve 80% power is at least 155.

## Results

### Demographic characteristics and antibody levels

**Figure 1:**
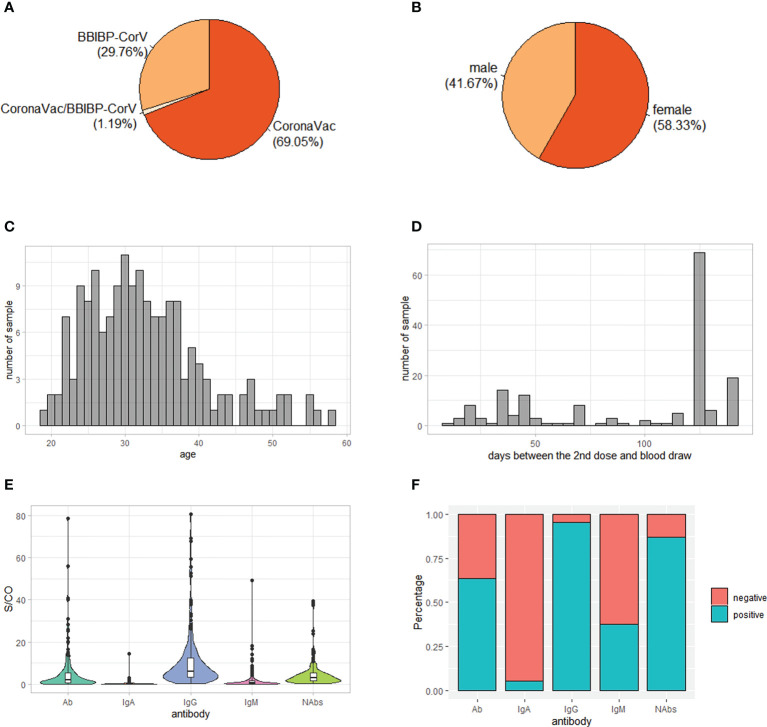
Demographic characteristics and antibody levels of the 168 study subjects. **(A)** Vaccine type distribution; **(B)** Sex ratio; **(C)** Age distribution; **(D)** Distribution of days between the 2nd dose and blood draw; **(E)** Level of IgG, IgM, IgA, total antibodies (Ab) and neutralizing antibodies (NAbs) S/CO: sample/cutoff. **(F)** Percentage of positive individuals for IgG, IgM, IgA, Ab and NAbs.

A total of 176 individuals who had received two doses of inactivated SARS-CoV-2 vaccine participated in our study. After removing close related individuals and poor genotyped individuals based on genome-wide genotyping data, 168 individuals were kept for further analysis. Of these, 116 were vaccinated two doses of CoronaVac vaccine, 50 were vaccinated two doses of BBIBP-CorV vaccine and two were vaccinated one dose of CoronaVac vaccine and one dose of BBIBP-CorV vaccine (**Figure 1A**). Female individuals were slightly more than male (58% vs. 42%) (**Figure 1B**). The majority of individuals were young, with a median age of 31.5 years (**Figure 1C**). The time from the second SARS-CoV-2 vaccination to blood draw was between 12 and 141 days, with nearly half of individuals (41%) being 126 days (**Figure 1D**).

Consistent with previous reports (12, 31), the serum antibody response to inactivated SARS-CoV-2 vaccines was mainly IgG, with the median level of IgG being 6.13 while the median levels of IgA and IgM being 0.20 and 0.56, respectively (**Figure 1E**). In addition, 95% of individuals was positive of IgG while only 5% and 38% of individuals was positive of IgA and IgM, respectively (**Figure 1F**). As expected, the level of Ab was positively correlated with levels of IgG, IgM and IgA (**Figure S1A**). On the other hand, the level of NAbs was positively correlated with level of IgG, suggesting that it was mainly IgG contributing to neutralizing effect of antibodies against SARS-CoV-2 (**Figure S1A**).

Differences of IgM and Ab levels were observed in different vaccine type groups (P = 6e-5 and P = 2e-4, respectively), with a higher level in BBIBP-CorV group than CoronaVac group, while IgG, IgA and NAbs levels had no significant differences among different vaccine type groups (P > 0.05) (**Figure S1B**). Furthermore, sex and age had no significant effect on antibody levels (**Figures S1C, D**). However, the time interval between the 2nd dose of immunization and blood draw was negatively correlated the levels of IgG, IgM, IgA and Ab, suggesting that the antibody levels might decline with time (**Figure S1D**).

### Genetic associations with antibody responses

To characterize the genetic association with antibody responses after SARS-CoV-2 inactivated vaccine immunization, we performed genome-wide association study of IgG, Ab and NAbs levels in linear regression models adjusting for covariates including: age, gender and the top five principal components from PCA. As for IgM and IgA, because their levels were low and a large proportion of individuals were negative, we categorized the individuals into positive group and negative group based on the seropositivity, and carried out genome-wide association tests in logistic regression models. Q-Q plots of the observed vs. expected -log_10_(P) indicated that there was no severe inflation in these statistical tests (**Figure S2**).

**Figure 2:**
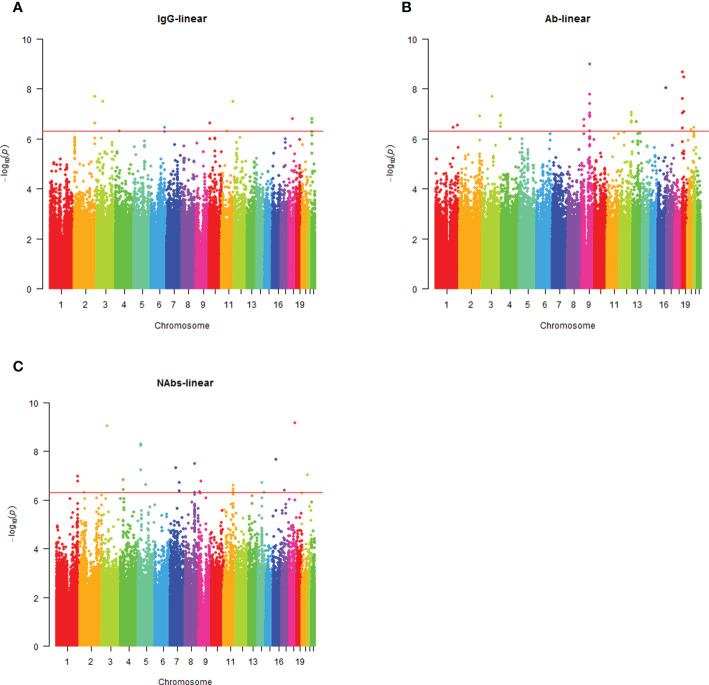
Manhattan plot summaries of GWAS results for antibody response. **(A)** Results for IgG level; **(B)** Results for Ab level; **(C)** Results for NAbs level.

Analysis of the genome-wide association data identified a total of 177 SNP associations with variations in antibody levels at P < 5e-7, corresponding 41 independent loci. Among these associations, 12 SNPs, corresponding eight independent loci were associated with IgG level (**Figure 2A**); 99 SNPs, corresponding 15 independent loci were associated with total antibodies level (**Figure 2B**); 66 SNPs, corresponding 18 independent loci were associated with neutralizing antibodies level (**Figure 2C**). The lead independent SNPs were listed in **Tables 1**–**3**. No SNPs were significantly associated with the seropositivity of IgM or IgA (**Figure S3**).

**Table 1:** Lead independent SNPs associated with IgG level.

SNP	Position	Alleles(Ref/Alt)	Gene	Location	Beta	SE	P
rs671398	2:219706616	A/G	–	intergenic	14.44	2.441	2.04E-08
rs12489684	3:67503800	C/T	SUCLG2	intron	14.35	2.465	3.23E-08
rs77592003	11:121406910	G/A	–	intergenic	16.82	2.894	3.39E-08
rs4799419	18:36510951	A/T	FHOD3	intron	13.94	2.539	1.59E-07
rs5994195	22:17197777	G/A	ADA2	intron	14.7	2.679	1.62E-07
rs10906319	10:13178143	T/G	MCM10	intron	10.58	1.956	2.37E-07
rs17072962	6:143713654	G/A	PHACTR2	intron	11.61	2.184	3.64E-07
rs80147812	11:58956910	G/A	GLYATL1	downstream	12.61	2.403	5.00E-07

**Table 2:** Lead independent SNPs associated with Ab level.

SNP	Position	Alleles(Ref/Alt)	Gene	Location	Beta	SE	P
rs117838393	16:82339985	G/A	–	intergenic	12.35	1.91	1.20E-09
rs72737661	9:84688251	G/C	NTRK2	intron	12.44	1.914	1.03E-09
rs146253416	19:1449555	G/A	APC2	upstream	12.3	1.935	2.16E-09
rs149813122	19:17557558	C/T	NIBAN3	downstream	12.17	1.943	3.48E-09
rs117838393	16:82339985	G/A	–	intergenic	11.04	1.818	9.22E-09
rs117118809	3:99677789	T/C	COL8A1	intron	10.71	1.814	2.13E-08
rs718784	19:18047613	G/T	RPS18P13	promoter	9.677	1.719	8.21E-08
rs180977908	12:116414208	T/G	–	intergenic	10.85	1.933	8.86E-08
rs12492145	3:191266812	G/C	UTS2B	downstream	10.58	1.905	1.19E-07
rs35463043	2:217021378	G/A	IGFBP-AS1	intron	11.22	2.026	1.27E-07
rs72715264	9:28773701	A/T	LINGO2	intron	11.05	2.019	1.71E-07
rs200076663	13:60294764	G/C	–	intergenic	9.647	1.778	2.16E-07
rs4543780	1:231038685	C/T	FAM89A	intron	9.113	1.698	2.86E-07
rs7269650	20:63071766	T/G	LINC01749	intron	9.332	1.756	3.64E-07
rs10798156	1:188009513	C/A	–	intergenic	9.123	1.717	3.65E-07
rs117744190	20:34517105	C/G	DYNLRB1	intron	11.35	2.151	4.35E-07

**Table 3:** Lead independent SNPs associated with NAbs level.

SNP	Position	Alleles(Ref/Alt)	Gene	Location	Beta	SE	P
rs80339225	18:59276950	T/C	RAX	upstream	10.64	1.619	7.19E-10
rs13094466	3:59957291	G/T	FHIT	intron	8.506	1.303	9.02E-10
rs1921111	5:30870751	G/A	–	intergenic	10.64	1.719	5.12E-09
rs2178487	5:30843681	T/A	–	intergenic	8.025	1.301	5.78E-09
rs755295	16:27794997	C/T	GSG1L	intron	9.44	1.599	2.17E-08
rs12548840	8:99885580	C/T	COX6C	intron	6.115	1.051	3.31E-08
rs115391410	7:55046103	T/C	EGFR	intron	9.572	1.668	4.86E-08
rs4630823	20:56534912	A/G	FAM209B	intron	7.386	1.318	9.36E-08
rs13104003	4:32053616	C/T	LINC02506	intron	7.726	1.405	1.54E-07
rs117761967	9:27576974	G/A	C9orf72	upstream	8.651	1.582	1.77E-07
rs7146742	14:65566216	G/A	FUT8	intron	5.177	0.9498	1.94E-07
rs3779197	7:98341212	C/T	BAIAP2L1	intron	9.422	1.731	2.02E-07
rs76017198	5:84708147	C/T	–	intergenic	7.867	1.457	2.45E-07
rs4457720	11:106621114	G/A	–	intergenic	6.239	1.157	2.56E-07
rs75953002	17:33615673	G/A	ASIC2	intron	8.387	1.585	4.08E-07
rs74609604	7:98178332	G/A	LMTK2	intron	8.979	1.7	4.29E-07
rs10977830	9:9515044	T/C	PTPRD	intron	9.693	1.842	4.69E-07

### Annotation of significant associated SNPs

Annotation of significant associated SNPs using Ensembl Variant Effect Predictor (VEP) (26) revealed that the majority of SNPs (86.49%) resided in the intron of genes and intergenic region, and none of SNPs resided in the exon of genes (**Figure S4**), suggesting that these SNPs might function as regulatory variants affecting gene expression. Thus, we prioritized the regulatory potential of significant SNPs using RegulomeDB (27), a database that annotates SNPs based on chromatin immunoprecipitation (ChIP)-seq, formaldehyde-assisted isolation of regulatory elements (FAIRE), and DNase I hypersensitive site data sets from the Encyclopedia of DNA Elements (ENCODE) project (28).

**Figure 3:**
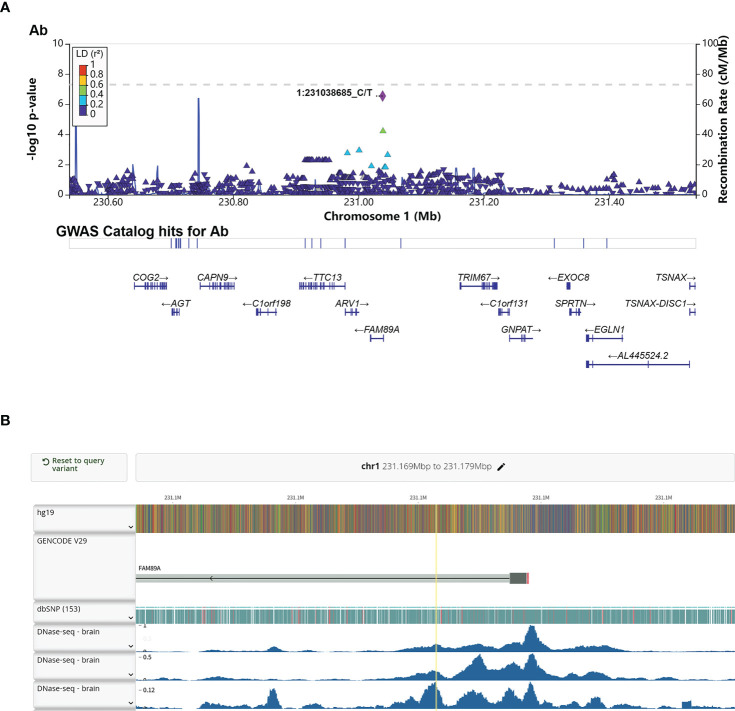
Annotation of the significant association SNP rs4543780. **(A)** LocusZoom plot showed the P value of the SNPs centering the lead SNP rs4543780, linkage disequilibrium degree, the recombination rate (top panel), SNP hits in GWAS catalogue (middle panel) and the genes in the region (bottom panel). 1:231038685_C/T indicated rs4543780 residing in chr1: 231038685 with reference allele of C and alternative allele of T; **(B)** DNase-seq peaks in the region of rs4543780 annotated by RegulomeDB. The yellow vertical line indicated the position of rs4543780.

Interestingly, rs4543780, an intronic SNP associated with total antibodies level (P = 2.86e-7, **Figure 3A** and **Table 2**), was ranked as 1f (**Figure S5**), which meant it was an eQTL and overlapped with transcription factor binding site or DNase peak. Indeed, it resided in DNase peak (**Figure 3B**) and GTEx data (32) showed that T allele of this variant was associated with lower expression of FAM89A gene in brain and muscle (**Table 4**), suggesting this variant might contribute to antibody response through regulating FAM89A gene expression. In addition, though the function of FAM89A gene has barely been investigated, this gene has been found to be upregulated in pathogen infections (33–35), indicating that it might be involved in immune response.

**Table 4:** Association between rs4543780 and FAM89A gene expression revealed by GTEx (V8) eQTL data.

Tissue	Gene Name	SNP	Effective allele	Beta	SE	P value
Brain_Caudate_basal_ganglia	FAM89A	rs4543780	T	-0.5751	0.0660	4.3255E-15
Brain_Nucleus_accumbens_basal_ganglia	FAM89A	rs4543780	T	-0.6206	0.0718	5.0347E-15
Brain_Cortex	FAM89A	rs4543780	T	-0.4653	0.0570	8.109E-14
Brain_Frontal_Cortex_BA9	FAM89A	rs4543780	T	-0.4634	0.0568	2.1099E-13
Brain_Anterior_cingulate_cortex_BA24	FAM89A	rs4543780	T	-0.4793	0.0617	2.7744E-12
Brain_Amygdala	FAM89A	rs4543780	T	-0.5346	0.0738	8.0559E-11
Brain_Putamen_basal_ganglia	FAM89A	rs4543780	T	-0.5169	0.0756	2.8562E-10
Brain_Hippocampus	FAM89A	rs4543780	T	-0.4899	0.0814	1.8018E-8
Brain_Substantia_nigra	FAM89A	rs4543780	T	-0.3855	0.0628	2.2684E-8
Brain_Hypothalamus	FAM89A	rs4543780	T	-0.3122	0.0570	2.1706E-7
Brain_Cerebellar_Hemisphere	FAM89A	rs4543780	T	-0.2178	0.0490	1.8106E-5
Muscle_Skeletal	FAM89A	rs4543780	T	-0.1630	0.0401	5.3924E-5

## Discussion

In this study, we identified 177 SNPs, corresponding to 41 independent loci, that were associated with IgG, total antibodies or neutral antibodies. Specifically, the intronic variant of FAM89A gene, rs4543780, which was associated with total antibodies level, was annotated as a potential regulatory variant affecting FAM89A gene expression.

To the best of our knowledge, this is the first genome-wide association study of antibody response to SARS-CoV-2 vaccine. Previously there were two candidate gene studies. Ragone et al. found HLA did not impact on short-medium-term antibody response to Pfizer-BioNTech BNT162b2 vaccine, which was a SARS-CoV-2 mRNA vaccine (36), though HLA variants have been shown to be associated with antibody response to several other pathogen vaccines (15–18, 37–39). Consistent of this research, we did not found associations between HLA variants and antibody response either. Another study by Colucci et al. found associations between allelic variants of the human IgH 3’ regulatory region 1 and the immune response to BNT162b2 mRNA vaccine (21). However, these variants did not pass the statistical threshold in our analysis, probably due to different kinds of vaccines, as we only had inactivated COVID-19 vaccine recipients available. On the other hand, our genome-wide analysis newly identified 177 SNPs, corresponding to 41 independent loci, that were associated with IgG, total antibodies or neutral antibodies.

Annotation of these significant associated SNPs revealed that none of SNPs resided in the exon of genes, with the majority of SNPs (86.49%) residing in the intron of genes and intergenic region, suggesting that these SNPs might function as regulatory SNPs of gene expression. Further annotation by RegulomeDB (27) prioritized the intronic SNP of FAM89A gene, rs4543780, as a potential regulatory SNP. This variant was significantly associated with total antibodies level. Moreover, ENCODE (28) data showed it resided in the open chromatin region and GTEx (32) data showed it was associated with FAM89A gene expression, suggesting that it might affect antibody response by regulating FAM89A gene expression. Though the function of FAM89A gene is not well studied, researches indicated that FAM89A gene, together with IFI44L gene, was capable of differentiating between bacterial and viral infections with high sensibility and specificity (33–35), suggesting that FAM89A gene might be involved in immune response. However, FAM89A had elevated expression in the blood of febrile children with bacterial infection rather than viral infection, which seemed to conflict with FAM89A gene being associated with antibody response to virus vaccines. One possible reason for this discrepancy might be tissue-specific gene expression regulation, as GTEx data showed rs4543780 was associated with FAM89A gene expression in brain and muscle instead of blood.

In addition to FAM89A gene, a number of genes implicated in COVID-19 and immune process were identified to harbor polymorphisms associated with antibody response to COVID-19 vaccines, such as ADA2 (Adenosine Deaminase 2), COX6C (Cytochrome C Oxidase Subunit 6C), FUT8 (Fucosyltransferase 8) and ASIC2 (Acid Sensing Ion Channel Subunit 2).

ADA2 gene encodes a member of a subfamily of the adenosine deaminase protein family that contributes to the degradation of extracellular adenosine, a signaling molecule that controls a variety of cellular responses. Serum increases of ADA2 activity has been described in patients with bacterial and viral diseases (40, 41), including individuals with SARS-CoV-2 infection and who recovered from infection (42). The deficiency of adenosine deaminase 2 (DADA2) is an autosomal recessively inherited disease that undergoes immune dysregulation including hypogammaglobulinemia, absent to low class-switched memory B cells, and inadequate response to vaccination (43). In our study, rs5994195 in the intron of ADA2 was identified to be associated with IgG level of COVID-19 vaccine immunization.

COX6C gene encodes component of the cytochrome c oxidase, the last enzyme in the mitochondrial electron transport chain which drives oxidative phosphorylation. COX6C is differentially expressed in multiple myeloma (MM) and is associated with MM prognosis (44). Multiple myeloma (MM) is a malignant proliferation of plasma cells, with the coexistence of a monoclonal component (M-component) plus impairment of normal immunoglobulin production, which are associated with increased risk of viral and bacterial infections (45). MM patients with COVID-19 show a longer duration to clinical improvement (46) and a higher risk of inpatient mortality (47). Moreover, COX6C is downregulated in patients with mild COVID-19 infection compared with controls but is upregulated in patients with severe COVID-19 compared with patients with mild illness (44). In our study, rs12548840 in the intron of COX6C was identified to be associated with NAbs level of COVID-19 vaccine immunization.

FUT8 gene encodes an enzyme belonging to the family of fucosyltransferases. Core fucosylation of IgG B cell receptor by FUT8 is required for antigen recognition and antibody production (48, 49). In addition, genome-wide association study revealed multiple SNPs in FUT8 gene had strong influences on the IgG glycosylation patterns (50, 51). In our study, rs7146742 in the intron of FUT8 was identified to be associated with NAbs level of COVID-19 vaccine immunization.

ASIC2 gene, also known as ACCN1 (Amiloride-Sensitive Cation Channel Neuronal 1), encodes the cation channel with high affinity for sodium, which is gated by extracellular protons and inhibited by the diuretic amiloride. The SNP rs28936 located in the 3’ UTR of ASIC2 gene is significantly associated with Multiple Sclerosis (MS) (52), an autoimmune disease that your immune system mistakenly attacks cells in the myelin and interrupts nerve signals from your brain to other parts of your body. In addition, an increase of ASIC2 mRNA was observed in the human autoptic brain tissue of MS patients and knockout of Asic2 resulted in a significant reduction in the clinical score in experimental autoimmune encephalomyelitis (EAE) mice model (53), highlighting the involvement of ASIC2 in the immune progress. In our study, rs75953002 in the intron of ASIC2 was identified to be associated with NAbs level of COVID-19 vaccine immunization.

The primary limitation of this study is the relatively small sample size, which might result in limited statistical power and excess false positive results. Besides, population stratification, vaccine types and the time from immunization to antibody detection could potentially bias the results. As conditions like smoking, hypertension and type 2 diabetes are associated with COVID-19 outcomes (54–56), and diseases such as schizophrenia and Alzheimer’s disease tend to be post-COVID-19 sequelae (57, 58), they may also be confounding factors for an immune reaction to SARS-CoV-2 vaccination. Therefore, further validation of our findings in a larger cohort will be needed.

In summary, we have identified a list of associated genetic variants contributing to inter-individual variation in antibody response after SARS-CoV-2 vaccination, which might inspire further genetic association researches and contribute to biological insights into vaccine response and better vaccine development.

## Data availability statement

The datasets presented in this study can be found in online repositories. The names of the repository/repositories and accession number(s) can be found in the article/**Supplementary Material**.

## Ethics statement

The studies involving human participants were reviewed and approved by The Institutional Review Board (IRB) of BGI-Shenzhen. The patients/participants provided their written informed consent to participate in this study.

## Author contributions

SX, YZ, and ZZ contributed to conception and design of the study. DS and SX were responsible for recruitment of vaccinated volunteers. PL and DS performed the experiments. PL analyzed the data. DS, WS, SS, XG, and JL helped to analyze and interpret the data. PL wrote the first draft of the manuscript. All authors contributed to the article and approved the submitted version.

## Funding

This work was supported by grants from the National Key Research and Development Program of China (2018YFC1200704, 2018YFA0900801) and the National Natural Science Foundation of China (31801087).

## Acknowledgments

We thank all the participants in this study.

## Conflict of interest

The authors declare that the research was conducted in the absence of any commercial or financial relationships that could be construed as a potential conflict of interest.

## Publisher’s note

All claims expressed in this article are solely those of the authors and do not necessarily represent those of their affiliated organizations, or those of the publisher, the editors and the reviewers. Any product that may be evaluated in this article, or claim that may be made by its manufacturer, is not guaranteed or endorsed by the publisher.

## Supplementary material

Supplementary Figure 1 Correlation between demographic characteristics and antibody levels. **(A)** Correlation between levels of different antibodies; **(B)** Antibody levels among different vaccine types; **(C)** Antibody levels between males and females. B: BBIBP-CorV, C: CoronaVac, BC: CoronaVac/BBIBP-CorV; **(D)** Correlation of antibody levels with age and interval from the 2nd dose to blood draw. * indicated P<0.05, ** indicated P<0.01, *** indicated P<0.001

Supplementary Figure 2 Q-Q plot plots of the expected (x-axis) and observed (y-axis) -log10 (P) in SARS-CoC-2 vaccine response GWAS. **(A)** Results for IgG level; **(B)** Results for Ab level; **(C)** Results for NAbs level; **(D)** Results for IgA positivity; **(E)** Results for IgM positivity

Supplementary Figure 3 Manhattan plot summaries of GWAS results for IgA and IgM. **(A)** Results for IgA positivity using logistic regression approach; **(B)** Results for IgM positivity using logistic regression approach.

Supplementary Figure 4 Distribution of the relative position of significant SNPs to genes.

Supplementary Figure 5 Distribution of the ranks of significant SNPs obtained from RegulomeDB. Red, green and blue indicated significant SNPs associated with Ab, IgG and NAbs level, respectively. The detail meaning of the rank represent could be found in the help page of RegulomeDB (https://regulome.stanford.edu/regulome-help/)
