# Supplementary figures and images for: Pilot genome-wide association study of antibody response to inactivated SARS-CoV-2 vaccines
Source: Front Immunol. 2022 Nov 14;13:1054147. doi: 10.3389/fimmu.2022.1054147 (PMC9704361; doi:10.3389/fimmu.2022.1054147)

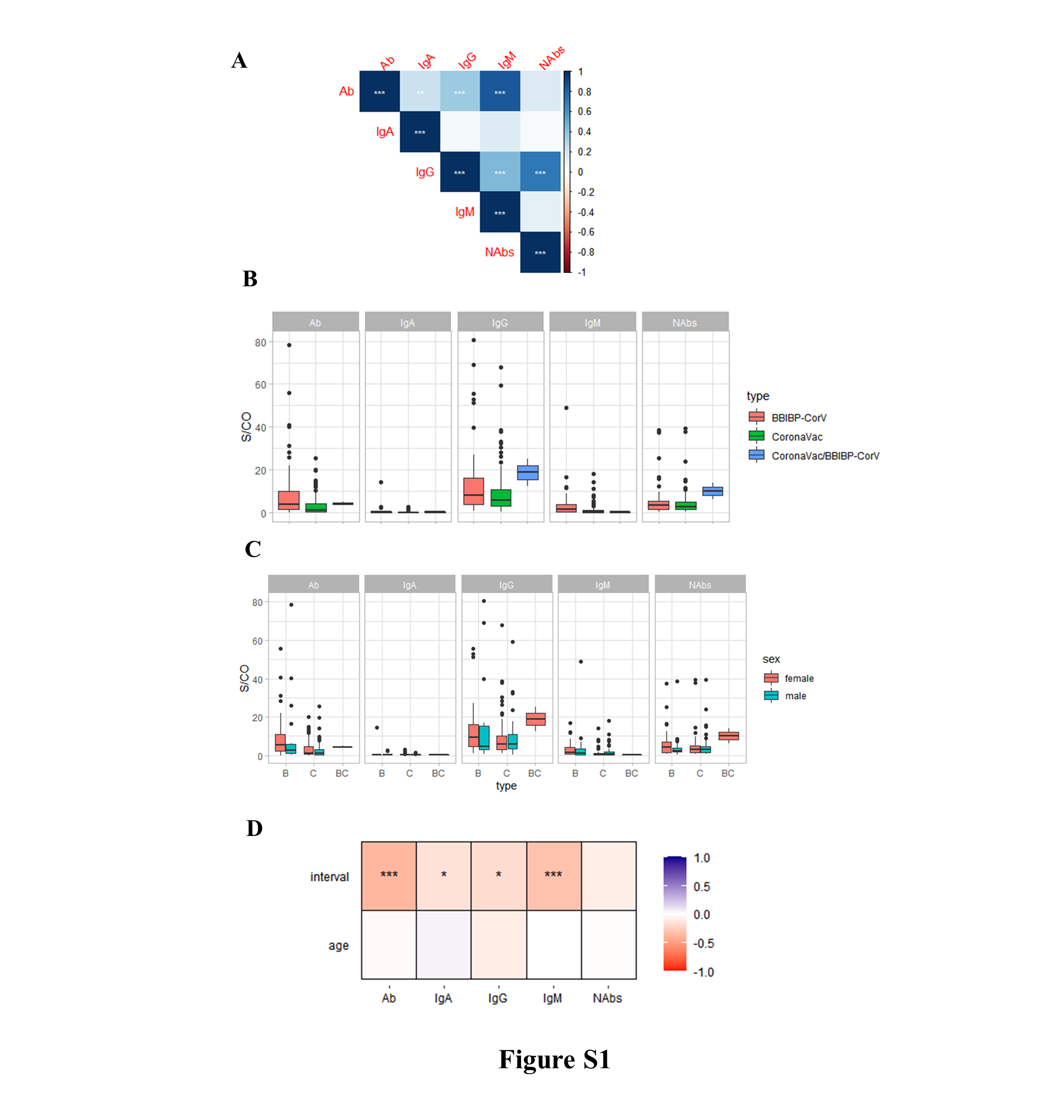

Supplement: Supplementary Figure 1 — Correlation between demographic characteristics and antibody levels. (A) Correlation between levels of different antibodies; (B) Antibody levels among different vaccine types; (C) Antibody levels between males and females. B: BBIBP-CorV, C: CoronaVac, BC: CoronaVac/BBIBP-CorV; (D) Correlation of antibody levels with age and interval from the 2nd dose to blood draw. * indicated P<0.05, ** indicated P<0.01, *** indicated P<0.001 [file Image_1.tif]

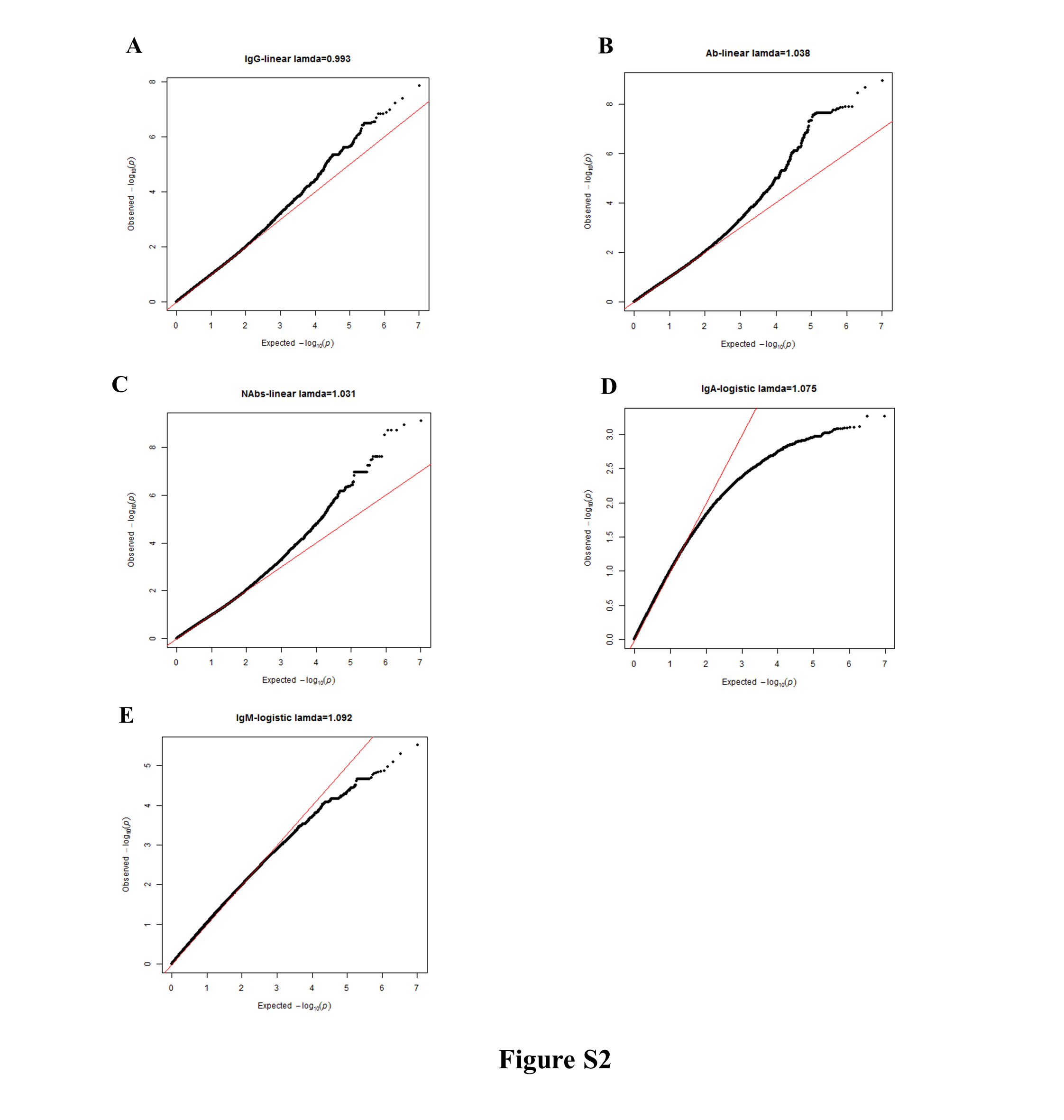

Supplement: Supplementary Figure 2 — Q-Q plot plots of the expected (x-axis) and observed (y-axis) -log10 (P) in SARS-CoC-2 vaccine response GWAS. (A) Results for IgG level; (B) Results for Ab level; (C) Results for NAbs level; (D) Results for IgA positivity; (E) Results for IgM positivity [file Image_2.tif]

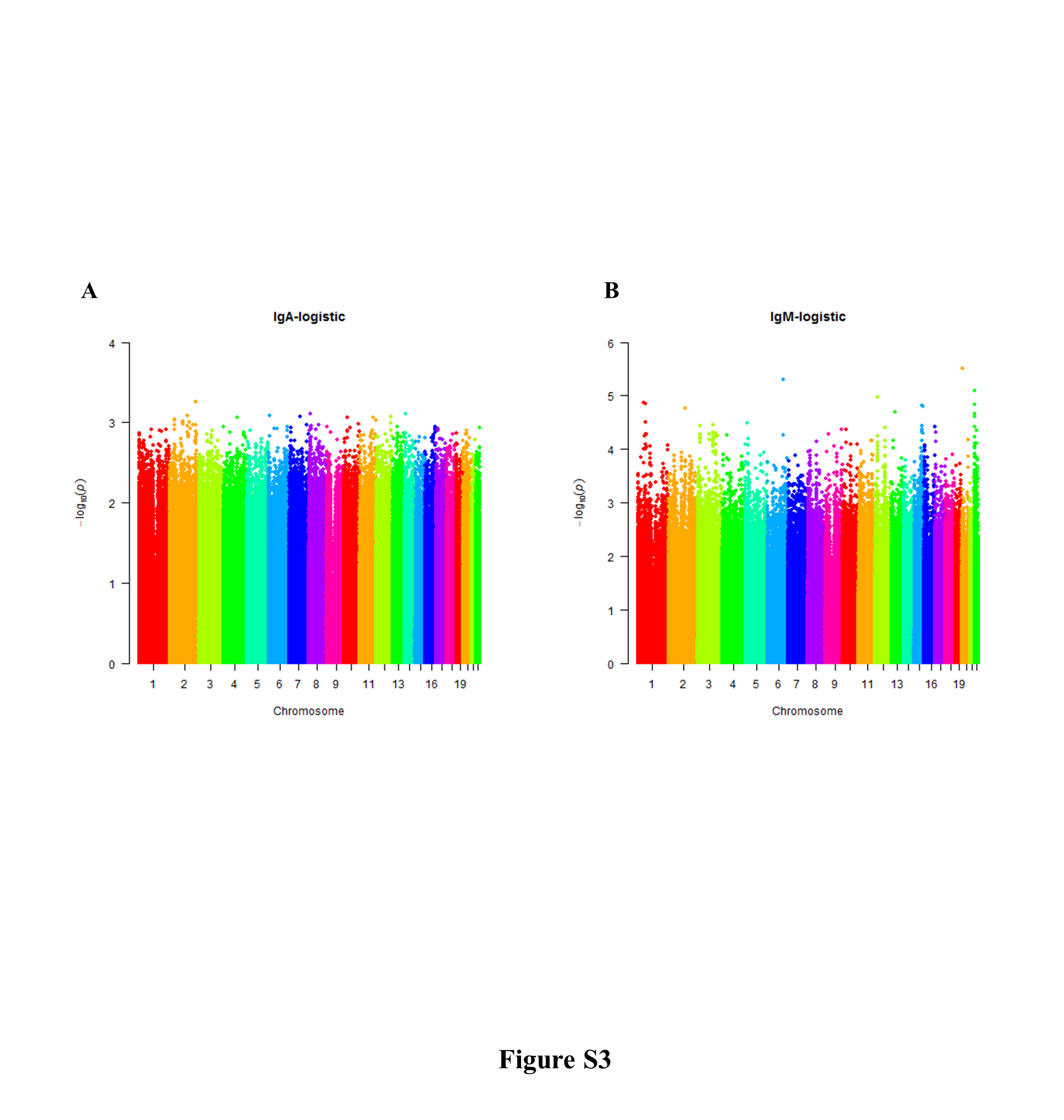

Supplement: Supplementary Figure 3 — Manhattan plot summaries of GWAS results for IgA and IgM. (A) Results for IgA positivity using logistic regression approach; (B) Results for IgM positivity using logistic regression approach. [file Image_3.tif]

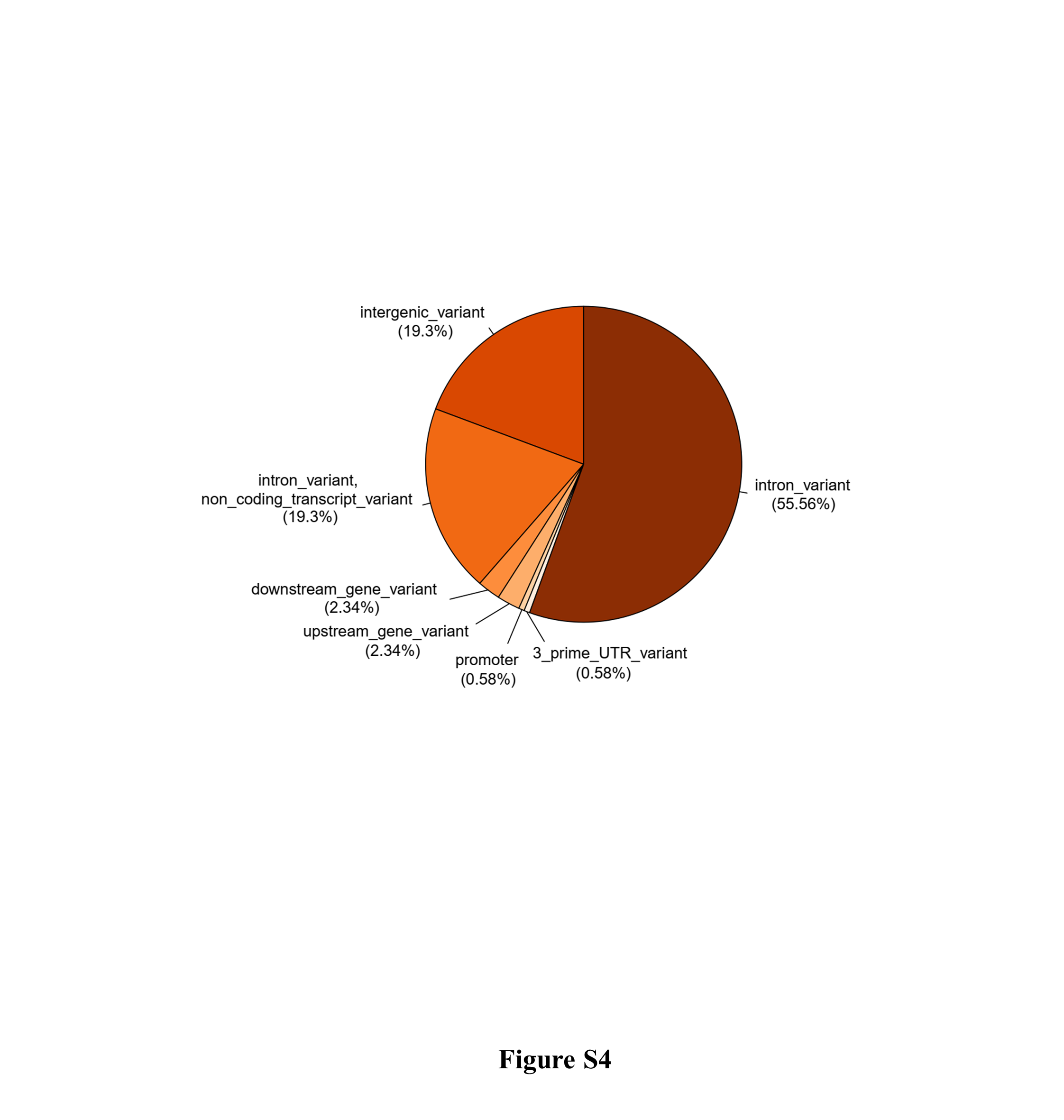

Supplement: Supplementary Figure 4 — Distribution of the relative position of significant SNPs to genes. [file Image_4.tif]

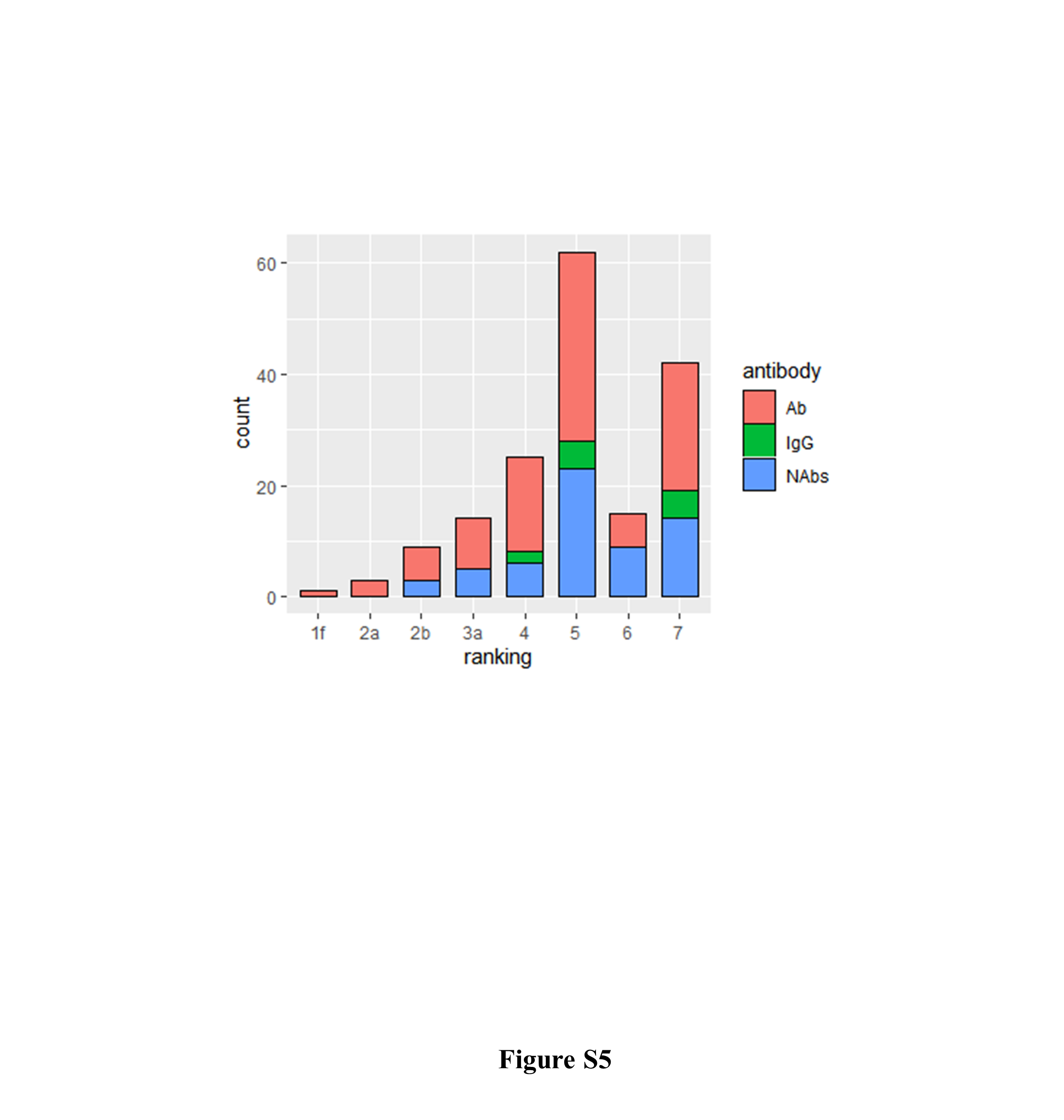

Supplement: Supplementary Figure 5 — Distribution of the ranks of significant SNPs obtained from RegulomeDB. Red, green and blue indicated significant SNPs associated with Ab, IgG and NAbs level, respectively. The detail meaning of the rank represent could be found in the help page of RegulomeDB (https://regulome.stanford.edu/regulome-help/) [file Image_5.tif]
